# Ten years of Israel’s organ transplant law: is it on the right track?

**DOI:** 10.1186/s13584-018-0232-1

**Published:** 2018-08-01

**Authors:** Jeffrey Zaltzman

**Affiliations:** 1 Division of Nephrology, St. Michael’s Hospital, Toronto, Ontario Canada; 2 Chief Medical Officer transplant, Trillium Gift of Life Network, Toronto, Ontario Canada; 3 University of Toronto, Toronto, Ontario Canada

**Keywords:** Brain death, Donation after cardiocirculatory death, Routine retrieval

## Abstract

The Israeli organ donor law was established in 2008. In the ensuing 10 years there have been some improvements in deceased donation and living donor rates and a reduction in the unethical practice of transplant tourism. There is, however, controversy regarding increased access to transplant for those who have been living donors, who are family members of deceased donors, or who have registered their intent to donate. The issue of routine retrieval versus obtaining consent for organ donation has also been raised. This commentary will address these issue, and propose some steps for improvement of the current Israeli organ donation system.

## Background

Israel’s organ transplant and brain-death law was passed in Israel in 2008 and fully implemented in 2010. It was developed as a response to the three major challenges to organ procurement and transplantation in Israel: 1) confusion regarding determination of death, 2) organ trafficking and unethical/illegal transplant tourism, and 3) the critical dearth of transplantable organs.

A major objective of the law, was an attempt to strengthen brain death determination to satisfy both medical and religious needs. This required voluntary training by the critical care physicians responsible for the determination of death by neurologic criteria. To date, the uptake by these physicians has been less than adequate. Unfortunately, brain death as a medical criterion for death is not uniformly accepted within the Israeli population, particularly amongst the ultra-Orthodox community.

A second objective was to stem the tide of transplant tourism as both an illegal and unethical means of recipients’ seeking solid organ transplantation. This has proven to have been successful, as demonstrated by the marked decrease in transplant tourism over the last 10 years [1]. This was partly accomplished by the reduction in financial disincentives to living organ donors. The state now reimburses the expenses associated with being a living donor. In fact, over the last eight years, live donation rates have doubled [1].

### Access to transplant

Perhaps the most controversial part of the law was a priority structure, favoring access to transplantation based on a point system. The highest priority was given to those whose first-degree relatives were deceased organ donors, or those who themselves had been a previous living donor. The next level of priority was given to those who choose to register as a donor. The last priority level is for individuals with first-degree family members who have registered as donors. This is in the context of an opt-in system whereby donor families must still provide consent in order to proceed with the organ donation, regardless of registration status.

In a recent IJHPR article [2], Berzon challenges the ethics of the point system and offers as a solution, an opt-out or routine retrieval policy [2]. In this commentary, I will try to reason that the ethical issues surrounding the organ donor law in Israel are not a major cause for concern. However, owing to the fact that Israel still falls short of its organ donor needs, other changes can be made to improve the organ donation rate in the country.

Although the point system only comes into effect after medical need is taken into account, Berzon argues that medical need alone should be the highest priority, and that access to transplantation must not be based on a predetermined hierarchy [2]. There is a concern that those who may not have access to media, or who may not be engaged in public awareness campaigns are at a disadvantage, as they would be less cognizant of the incentivized donor registration program.

It should be noted however, that in all transplant allocation policies around the world, societal factors are often employed ahead of medical need. One example illustrating this point is that uniformly, allocation systems for kidney transplants give higher priority to pediatric recipients versus their adult counterparts. In addition, patients with liver failure secondary to alcoholic cirrhosis are often obligated to wait a 6-month sobriety period before acceptance onto a wait list for liver transplantation [3]. There are no compelling medical reasons for either of these allocation practices.

In the general population, the likelihood of requiring a life-saving organ transplant is fivefold greater than the chance of being a deceased organ donor. This imbalance between supply and demand means that transplantation is highly dependent upon an adequate supply of both deceased and living organ donors. To date, no jurisdiction has been able to meet the need of an abundant supply of transplantable organs. Thus, different countries have had varying approaches to trying to address the imbalance. In the Israeli system, registration as a donor increases a person’s priority if he/she were ever to need a future transplant. In the absence of an adequate supply of organ donors (those willing to donate), the likelihood of receiving a transplant diminishes.

An analogy to organ donor registration is childhood vaccination policy. A child who is vaccinated may achieve some future health advantage. However, unless vaccination occurs within the vast majority of the population, preventable communicable diseases will still occur, and even a vaccinated child may still be at risk if immunity doesn’t develop. Conversely, parents who choose not to vaccinate their children may still be afforded protection so long as there is enough herd immunity (vaccination rates close to 100%). Similarly, if registration for organ donation ever approached levels nearing 100%, then the priority point system would become irrelevant, and organ Israeli donation rates could be amongst the highest in the world.

### Jurisdictional scan

When the Israeli organ donor law first came into existence in 2008 and implemented in 2010, there had been a lot of public and media attention. However, over time there has been less awareness and donor registration rates have been flat. The success of countries such as Spain is not based on an opt-out, or routine retrieval policy, but rather on a very well-resourced, well-organized organ donation community within that country [4]. Organ donation is embedded within the Spanish culture. The success of the Spanish model is based on ongoing communication and media attention that is afforded to transplantation and organ donation [4].

Within Israel’s ultra-orthodox community, there are many who do not accept brain death as a definition of death. This issue became very apparent with the establishment of the new brain death determination laws [5]. However, Israel’s challenges with the acceptance of brain-death are not unique. Even in countries such as the United States and Canada where there is a clearer distinction between church and state, familys’ religious beliefs often challenge the medical dogma of neurological determination of death [6].

A shift in policy from obtaining family consent to routine retrieval as posited by Berzon [2] is unlikely to succeed in Israel. First, in most jurisdictions where opt-out or routine retrieval is the law, physicians still obtain consent from family members before proceeding with organ donation. Only in Austria is there a true “hard” opt-out policy. In practice, unless otherwise stated, organ donation is presumed, and organ procurement occurs without next-of-kin-consent. It seems inconceivable that, based on the above stated views on brain death, that the ultra-orthodox community would be accepting of routine referral in the absence of consent for donation.

The United States does not practice routine referral and like Israel, requires consent from next-of-kin. The US has a donation rate of ~ 28–30 donors per million, which is better than the majority of jurisdictions with routine referral policies. Approximately 50% of its population are registered as organ donors, and in some states this number exceeds 85% (https://www.organdonor.gov/index.html).

### Solutions

The current point system has face validity and some of its key features should be maintained. At the same time, the ethical issues raised by Berzon should be given serious consideration and the point system should be modified accordingly.

In North America, like Israel, priority for access to transplant is given to those who have been previous living donors. This should be the highest priority within the point system. Priority to those who have registered as donors, or those with family members who have been organ donors, should be maintained.

I would, however, consider eliminating priority to individuals whose first degree-relatives have registered. This current paradigm can be challenged. Why a first degree relative and not a best friend? What is the relationship between a potential recipient who would get priority and their first degree relative?

Leaving aside the point priority, there is room for improvement in Israel’s organ donation system.

First, there needs to be a societal culture shift embracing organ donation. An intensive public campaign which had proved to be successful in the past needs to be reinvigorated and continued. This would involve reengagement of all communities.

Second, critical care physicians need to ensure that organ donation is an integral part of the end-of-life care that they provide. Skilled organ donation specialists can be employed and provide expertise across donor hospitals. 100% of critical care doctors need to have expertise in brain-death determination.

Third and most important, Israel needs to implement the practice of donation after cardiocirculatory death (DCD). Not only will this increase the organ donor supply, but it may help in part circumvent the issues surrounding neurological death determination in some religious communities.

In Ontario Canada with a population of 13.5 million, DCD accounts for approximately 33% of all deceased organ donors and has resulted in an additional 2000 transplants since its implementation in 2006. During this same period, donation after brain death criteria also increased [7]. Since 2002, organ donation rates in this province have increased from 12 per million to 26 per million.

## Conclusion

Israel has made tremendous improvements in the 10-year history of the 2008 organ donation laws. Some of these changes were bold and not without controversy. The organ donation system in Israel continues to face significant challenges. There is room for improvement without the need to abandon the current structure. Jurisdictions that have seen significant improvements in organ donation rates have had major shifts culture and compelling resource allocation.
